# Effects of *OsNRT2.3b* transgenosis on lettuce antioxidant capacity and nitrogen metabolism under low nitrogen

**DOI:** 10.1371/journal.pone.0352238

**Published:** 2026-07-01

**Authors:** Yuxuan Qian, Xue Liu, Baoju Wang, Ning Liu, Dayong Li, Zhanhui Wu, Jing Tong

**Affiliations:** 1 Key Laboratory of Urban Agriculture (North) of Ministry of Agriculture, Beijing Vegetable Research Center, Beijing Academy of Agriculture and Forestry Science, Beijing, China; 2 State Key Laboratory of Vegetable Biobreeding, Beijing Vegetable Research Center, Beijing Academy of Agriculture and Forestry Science, Beijing, China; 3 National Engineering Research Center for Vegetables, Beijing Vegetable Research Center, Beijing Academy of Agriculture and Forestry Science, Beijing, China; University of Agriculture in Krakow, POLAND

## Abstract

Nitrate (NO_3_^-^) plays dual roles in vascular plants, functioning as both an essential macronutrient and a phytoactive signaling compound. Research indicates that the high-affinity NO_3_^-^ transporter OsNRT2.3b orchestrates fundamental agronomic traits, including biomass accumulation, grain production, and nitrogen (N) metabolism efficiency in cultivated rice. In this study, transgenic lettuce lines of *OsNRT2.3b* were generated, from which four lines with high *OsNRT2.3b* expression (OE20−5, OE21−7, OE21−11, and OE21−13) were selected and subjected to normal (CK) and low nitrogen (LN) treatments for 12 d. The results indicate that *OsNRT2.3b* transgenosis increased chlorophyll and glutathione (GSH) contents, as well as the activities of superoxide dismutase (SOD), peroxidase (POD), and catalase (CAT), in lettuce. However, *OsNRT2.3b* transgenosis had little effect on certain agronomic traits (leaf number, plant height, and leaf dimensions) of lettuce. Compared to the wild-type (WT), *OsNRT2.3b* transgenosis substantially enhanced the contents of NO_3_^-^, ammonium (NH_4_^+^), amino acids, soluble protein, and the activities of NO_3_^-^ reductase (NR), nitrite (NO_2_^-^) reductase (NiR), glutamine synthetase (GS), and glutamate synthetase (GOGAT) in lettuce. Thus, *OsNRT2.3b* transgenosis improves the antioxidant capacity, photosynthetic level, and N use efficiency of lettuce. This research provides theoretical references for improving the N utilization efficiency of lettuce and enhancing its antioxidant capacity.

## Introduction

As a fundamental macronutrient, nitrogen (N) is indispensable for higher plant physiology, serving as a critical component in the biosynthesis of vital cellular constituents, including proteins, nucleic acids, photosynthetic pigments, and enzymatic cofactors [1–3]. Among various N sources, nitrate (NO_3_^-^) plays dual roles as both a primary nutrient and developmental regulator in plants [4,5]. Most NO_3_^-^ obtained by plants from soil is actively transported through a group of NO_3_^-^ transporters (NRTs) [6]. Plants have evolved both a low-affinity transport system (LATS, effective at >1 mM) and a high-affinity transport system (HATS, active at <1 mM) to accommodate varying environmental NO_3_^-^ concentrations [4]. The NRT family, categorized into NRT1/NPF, NRT2, and NRT3 subfamilies [7], exhibits functional specialization: NRT1 proteins mediate LATS; NRT2 proteins mediate HATS [8]; and NRT3 members modulate NRT2 activity for efficient high-affinity transport [9]. Beyond NO_3_^-^ assimilation, these transporters influence various developmental and stress adaptation processes [10,11].

Within rice N regulatory pathways, the *OsNRT2.3* gene serves as a crucial determinant of plant development, productivity, and N assimilation efficiency. This gene encodes a high-affinity NO_3_^-^ transport protein with two alternatively spliced variants: OsNRT2.3a and OsNRT2.3b [12,13]. In rice Nipponbare (*Oryza sativa* ssp. japonica), the level of *OsNRT2.3b* is usually higher than *OsNRT2.3a*. OsNRT2.3b is a high-affinity NO_3_^-^ transporter located on the plasma membrane. The genetic enhancement of *OsNRT2.3b* expression has been shown to significantly boost N acquisition and utilization efficiency across varying N regimes [12]. Remarkably, under phosphorus-deficient conditions, *OsNRT2.3b* overexpression confers a substantial 44% increase in grain production [14].

As a nutritious member of the Asteraceae family, lettuce (*Lactuca sativa* L.) is cultivated as an annual/biennial crop, providing valuable vitamins, minerals, and dietary fiber [15]. This leafy vegetable, prized for its delicate texture and palatability, exhibits consistent growth patterns and permits intensive cultivation cycles, contributing to its widespread agricultural production throughout China [16]. N management represents a critical factor in lettuce cultivation, as proper N fertilization directly impacts both productivity and quality parameters [17]. Insufficient N availability negatively affects crop performance by impairing photosynthetic efficiency and restricting overall plant growth [18]. The prolonged application of N fertilizer not only decreases crop yields but also results in soil acidification, hindering the progress of green agriculture [19,20]. Therefore, improving the utilization efficiency of N fertilizer has become a necessary requirement for the sustainable development of green agriculture.

The functions of *OsNRT2.3b* have been validated in rice. Thus, in this study, *OsNRT2.3b* transgenic lines were generated in lettuce, and four lines with high *OsNRT2.3b* expression (OE20−5, OE21−7, OE21−11, and OE21−13) were selected for further analysis under normal (CK) and low nitrogen (LN) conditions. This research aims to offer theoretical references for improving nitrogen utilization efficiency in lettuce and increasing its yields.

## Materials and methods

### Plant materials

Experiments were conducted at the Vegetable Research Institute of Beijing Academy of Agricultural and Forestry Sciences (116^◦^29′ E, 39^◦^94′ N). The lettuce cultivar Italy was used as the experimental material.

### The genetic transformation of *OsNRT2.3b*

The CDS sequence of *OsNRT2.3b* was cloned into the pEZR(K)-LC vector. The corresponding primer sequences were listed in S1 Table. The constructs were transformed into wild-type (WT) lettuce (Italy) using the agrobacterium-mediated cotyledon infection method. Experimental methods were described previously [21]. Specifically, lettuce seeds were first disinfected by soaking in 75% ethanol for one minute, followed by three washes. Subsequently, they were treated with a 30% bleach solution for 15 minutes and rinsed aseptically three times. The sterilized seeds were then placed on Murashige and Skoog (MS) solid medium and cultivated in a light incubator at 25 °C under a 16-hour photoperiod (16-hour light/8-hour dark cycle). After five days of growth, when the cotyledons had just unfolded, they were carefully excised and immersed in a 1/2 MS suspension containing *Agrobacterium tumefaciens* EHA105 for 18 minutes. The cotyledons were then transferred to co-culture medium MS1 (MS supplemented with 0.5 mg/L 6-benzylaminopurine and 0.1 mg/L naphthylacetic acid), covered with tinfoil, and incubated under the same light and temperature conditions (25 °C, 16-hour light/8-hour dark cycle) for three days.

Following co-culture, the leaves were transferred to differentiation medium MS2 (MS containing 0.5 mg/L 6-benzylaminopurine, 0.1 mg/L naphthylacetic acid, 50 mg/L kanamycin, and 50 mg/L timentin) and cultured for two to four weeks under identical conditions (25 °C, 16-hour light/8-hour dark cycle) to promote the growth of kanamycin-resistant seedlings. Once the seedlings exhibited sufficient root development, they were transplanted into MS4 rooting medium (MS with 50 mg/L timentin) before being transferred to soil. Then, generating *OsNRT2.3b* transgenic lines, the transformants and their offspring were screened, testing the inserted fragment (genomic DNA was extracted and subsequently amplified by PCR) and the expression level of *OsNRT2.3b*.

### Experimental treatments of transgenic lines and wild-type lettuce

T3 homozygous lines and the WT were selected for subsequent assays. Lettuce seedlings were cultivated in a peat-based growth medium (peat/vermiculite/perlite = 2:1:1) and nourished with Hoagland solution. When the lettuce sprouted seven true leaves, the plants were subjected to normal (CK, 11.5 mM NO_3_^-^) and low nitrogen (LN, 1.15 mM NO_3_^-^) treatments (irrigated with Hoagland nutrient solution and adjusted to the aforementioned NO_3_^-^ concentrations every three days). The NO_3_^-^ concentrations were designed with reference to previous publications [22,23]. After 12 d, the phenotypes were analyzed, and the physiological indices were determined.

### Phenotypic analysis and determination of physiological indices

Morphological parameters, including plant height and leaf dimensions (length and width), were recorded using standard measuring rulers. Chlorophyll quantification was performed through ethanol-based extraction followed by colorimetric analysis [24]. Commercially available assay kits (Solarbio, Beijing, China) were employed to quantify various biochemical parameters following the manufacturer’s instructions. These included antioxidant enzyme activities [superoxide dismutase (SOD), peroxidase (POD), and catalase (CAT)], oxidative stress markers [malondialdehyde (MDA) and glutathione (GSH) levels], N metabolites (NO_3_^-^, NH_4_^+^, amino acids, and soluble proteins), and key N metabolism enzymes [NO_3_^-^ reductase (NR), NO_2_^-^ reductase (NiR), glutamine synthetase (GS), and glutamate synthase (GOGAT)]. The corresponding catalogue numbers were listed in S2 Table.

### Expression analysis by qRT-PCR

The expression level of *OsNRT2.3b* was determined using quantitative real-time PCR (qRT-PCR). Specifically, RNA extraction was performed on plant samples using the FastPure Universal Plant Total RNA Isolation Kit (Vazyme, Nanjing, China). For cDNA synthesis, 1 µg of purified total RNA was processed using the HiScript III 1st Strand cDNA Synthesis Kit (+gDNA wiper) from the same manufacturer. qRT-PCR was conducted on a Bio-Rad CFX Opus 96 instrument (Hercules, CA, USA) to determine cycle threshold (C_t_) values. The reaction mixture (20 µL total volume) contained 10 µL of 2 × SYBR Green Master Mix (TOYOBO, Osaka, Japan), 0.5 µL each of forward and reverse primers, and 2 µL cDNA template, with the remaining volume completed by nuclease-free water. The experimental design incorporated triplicate biological replicates, using *18S rRNA* as an internal control. The 2^−∆∆Ct^ method was used to calculate the relative gene expression levels [25].

### Statistical analysis

Statistical analyses were performed using PASW Statistics 18 software (SPSS Inc., Chicago, IL), with post hoc comparisons evaluated through both LSD and Waller–Duncan tests to assess treatment differences. All experimental data were processed and visualized using Microsoft Excel 2016 (Microsoft Corp., Redmond, WA). Different letters in the figures indicate significant differences between the treatments (*p* < 0.05).

## Results

### PCR and qRT-PCR verifcation of the inserted fragment

**Fig 1:**
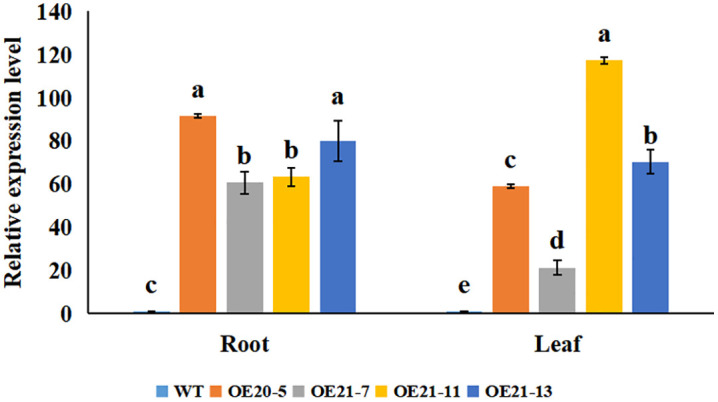
qRT-PCR analysis of *OsNRT2.3b.* The relative expression levels were calculated using the 2^−∆∆Ct^ method with three biological replicates; *18S rRNA* was used as the reference gene. Vertical bars indicate standard deviations, and different lowercase letters above the bars indicate significant differences (*p* < 0.05).

Transgenic lettuce lines overexpressing OsNRT2.3b were generated, and lines exhibiting high levels of transgene expression were subsequently selected from the T3 homozygous lines. When the plants have grown to 14 d, the inserted fragments were tested. Compared to the wild-type (WT) plants, the OsNRT2.3b expression levels in the four lines (OE20−5, OE21−7, OE21−11, and OE21−13) were significantly elevated (Fig 1). Genomic DNA was then extracted from these lines to verify the presence of the inserted fragment. PCR analysis confirmed successful integration in all selected transgenic lines (S1 Fig).

### Analysis of growth of wild-type and transgenic lettuce under normal and low nitrogen

**Fig 2:**
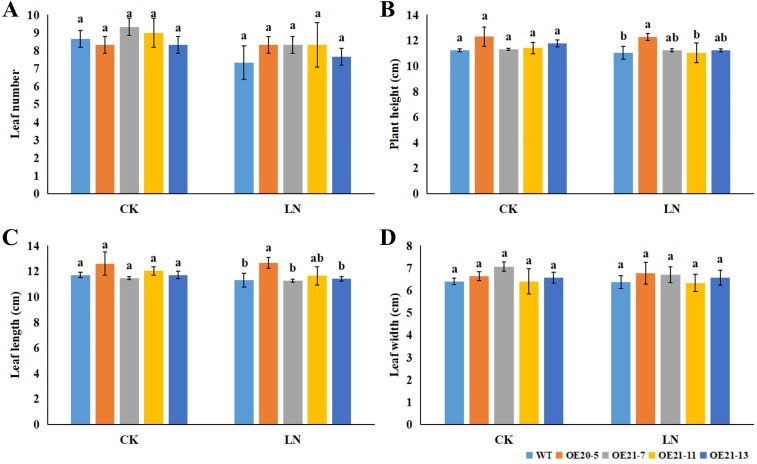
Analysis of key agronomic traits of wild-type (WT) and transgenic lettuce under normal (CK) and low nitrogen (LN) conditions. **(A)** Leaf number. **(B)** Plant height. **(C)** Leaf length. **(D)** Leaf width. The plant height, leaf length, and leaf width were measured using a ruler. Error bars represent the standard error of three replicates, and different lowercase letters above the bars indicate significant differences (*p* < 0.05).

OE20−5, OE21−7, OE21−11, and OE21−13 were selected for further analysis under normal (CK) and low nitrogen (LN) conditions. Then, the main agronomic traits (number of leaves, plant height, leaf length, and width) were examined. The analysis showed no statistically significant variation between the transgenic lines and the WT in most of these traits, indicating that *OsNRT2.3b* transgenosis has little effect on lettuce growth (Fig 2).

### Antioxidant capacity and chlorophyll level of wild-type and transgenic lettuce under normal and low nitrogen

**Fig 3:**
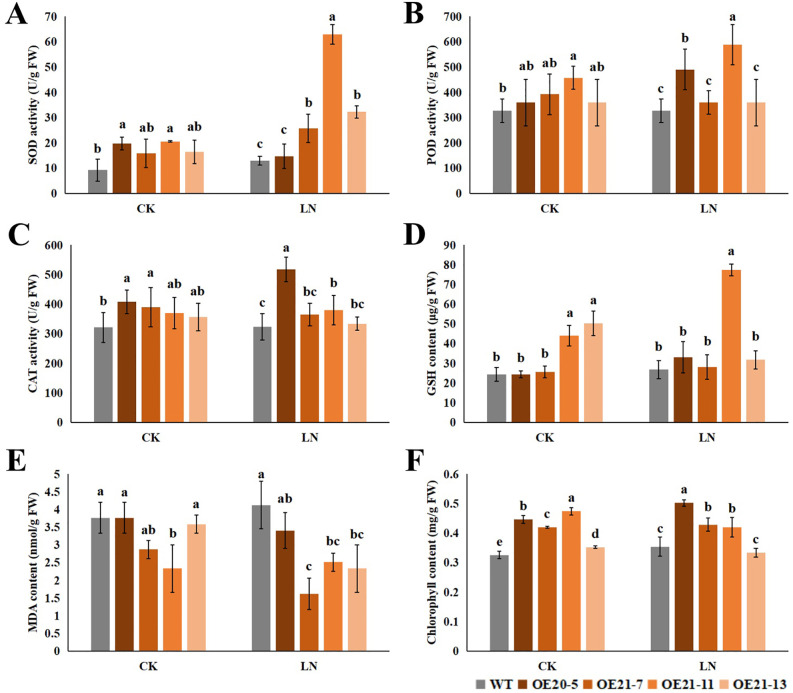
Antioxidant capacity, oxidative stress markers and chlorophyll levels of wild-type (WT) and transgenic lettuce under normal (CK) and low nitrogen (LN) conditions. **(A)** Superoxide dismutase (SOD) activity. **(B)** Peroxidase (POD) activity. **(C)** Catalase (CAT) activity. **(D)** Glutathione (GSH) content. **(E)** Malondialdehyde (MDA) content. **(F)** Chlorophyll content. Error bars represent the standard error of three replicates, and different lowercase letters above the bars indicate significant differences (*p* < 0.05).

Antioxidant capacity analysis showed that the transgenic lines exhibited significantly higher antioxidant enzyme activity [superoxide dismutase (SOD), peroxidase (POD), and catalase (CAT)] compared to WT plants (Fig 3A-C). Additionally, oxidative stress markers were assessed, revealing 4.59%–188.95% elevated glutathione (GSH) levels (Fig 3D) and reduced malondialdehyde (MDA) accumulation (Fig 3E) in transgenic lines. These findings suggest improved reactive oxygen species (ROS) scavenging capacity and diminished oxidative damage in transgenic lettuce. In addition, compared to WT plants, the chlorophyll content of transgenic lines was increased by 6.06%–42.86% (Fig 3F). These findings demonstrate that *OsNRT2.3b* transgenosis enhances LN stress tolerance in lettuce by enhancing antioxidant enzyme activity, promoting the synthesis of antioxidants, and maintaining photosynthetic efficiency.

### Nitrogen metabolism of wild-type and transgenic lettuce under normal and low nitrogen

**Fig 4:**
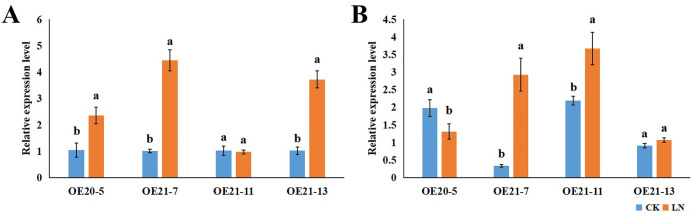
qRT-PCR analysis of *OsNRT2.3b* under normal (CK) and low nitrogen (LN) conditions. (A) *OsNRT2.3b* expression level in the root. (B) *OsNRT2.3b* expression level in the leaf. The relative expression levels were calculated using the 2^−∆∆Ct^ method with three biological replicates; *18S rRNA* was used as the reference gene. Vertical bars indicate standard deviations, and different lowercase letters above the bars indicate significant differences (*p* < 0.05).

As shown in Fig 4A, in the roots of OE20−5, OE21−7, and OE21−13, LN stress significantly upregulated *OsNRT2.3b* expression, with a similar effect observed in the leaves of OE21−7 and OE21−11 (Fig 4B). The analysis indicated that *OsNRT2.3b* can respond to LN stress in lettuce.

**Fig 5:**
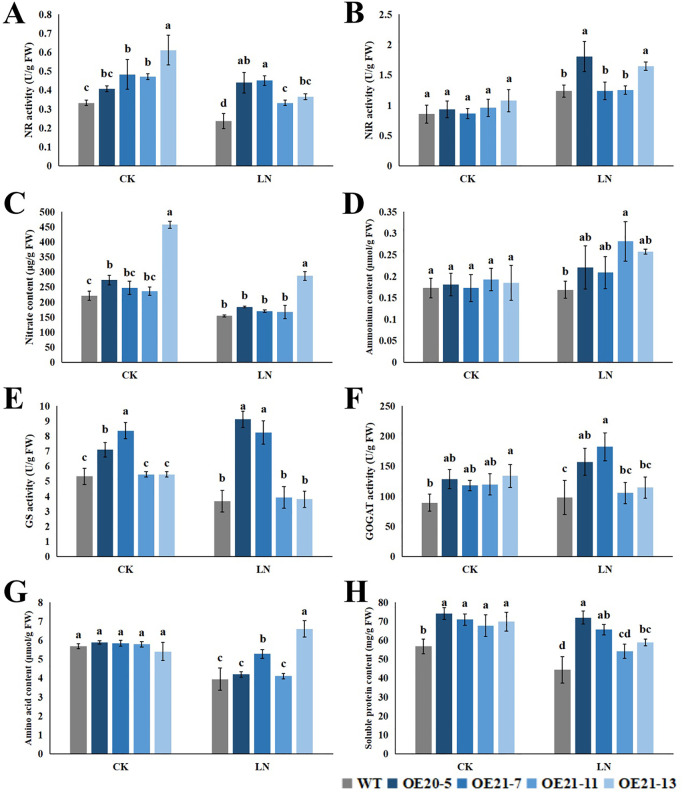
Nitrogen (N) metabolism levels of wild-type (WT) and transgenic lettuce under normal (CK) and low nitrogen (LN) conditions. **(A)** NO_3_^-^ reductase (NR) activity. **(B)** NO_2_^-^ reductase (NiR) activity. **(C)** NO_3_^-^ content. **(D)** Ammonium (NH_4_^+^) content. **(E)** Glutamine synthetase (GS) activity. **(F)** Glutamate synthetase (GOGAT) activity. **(G)** Amino acid content. **(H)** Soluble protein content. Error bars represent the standard error of three replicates, and different lowercase letters above the bars indicate significant differences (*p* < 0.05)..

Compared to WT plants, NO_3_^-^ reductase (NR) and NO_2_^-^ reductase (NiR) activities of transgenic lines were increased, especially under LN treatment (Fig 5A and B). For example, under LN treatment, the NR activity of the four transgenic lines was increased by 83.33%, 87.50%, 37.50%, and 50.00%. Furthermore, the NO_3_^-^ content of OE21−13 was higher than that of the WT (Fig 5C). Under LN treatment, ammonium (NH_4_^+^) contents were improved by 29.41%, 23.53%, 64.71%, and 52.94%, respectively (Fig 5D), and amino acid contents were increased by 6.35%, 34.01%, 4.06%, and 67.51%, respectively, compared to the WT (Fig 5G). Compared to the WT, soluble protein contents, glutamine synthetase (GS), and glutamate synthetase (GOGAT) activities were elevated in transgenic lines (Fig 5E,F,H). These findings demonstrate that *OsNRT2.3b* transgenosis boosts N metabolism in lettuce.

## Discussion

Nitrogen (N) fertilizers constitute a significant portion of energy consumption in agriculture [26]. Plant roots primarily absorb two inorganic N forms from the soil: ammonium (NH_4_^+^) and nitrate (NO_3_^-^) [27]. NH_4_^+^ is readily taken up by NH_4_^+^ transporters and directly metabolized into N. In contrast, NO_3_^-^ uptake depends on NO_3_^-^ transporters (NRTs), followed by its stepwise reduction to NH_4_^+^ via NO_3_^-^ reductase (NR) and NO_2_^-^ reductase (NiR) before assimilation [28]. The NRT family, essential for NO_3_^-^ uptake and translocation, plays a crucial role in regulating plant growth, development, and stress tolerance [29]. In rice, the high-affinity NO_3_^-^ transporter OsNRT2.3b plays a vital role in NO_3_^-^ uptake and assimilation [30,31]. Therefore, in this study, *OsNRT2.3b* transgenic lines were generated in lettuce and further analyzed, providing theoretical references for improving N utilization efficiency and offering novel directions for the molecular breeding of lettuce with improved yields.

When plants are exposed to abiotic stresses, various reactive oxygen species (ROS) are generated [32]. N stress, as a common abiotic stress factor, significantly impairs plant growth and induces various morphological and physiological disorders [33]. Both N deficiency and excess disrupt normal plant metabolic processes, with N deprivation particularly inducing oxidative stress [34]. Under such conditions, ROS accumulation triggers toxic redox reactions, causing cellular damage through lipid peroxidation, DNA impairment, and malondialdehyde (MDA) production [35]. To mitigate oxidative damage, plants enhance their antioxidant defense system by upregulating key enzymes, including superoxide dismutase (SOD), peroxidase (POD), and catalase (CAT) [36]. Consequently, MDA content and SOD, POD, and CAT activities serve as reliable biomarkers for assessing plant stress tolerance [37]. Moreover, glutathione (GSH), as an antioxidant, can enhance the antioxidant capacity of plants [38]. NRTs play significant roles in plant stress response [29]. In this study, low nitrogen (LN) treatment moderately increased SOD and CAT activities in wild-type (WT) plants compared with the normal-nitrogen control (CK). *OsNRT2.3b* transgenosis markedly increased the SOD, POD, and CAT activities of lettuce, especially under LN treatment. Meanwhile, MDA levels were reduced compared to WT. It follows that OsNRT2.3b improved the antioxidant capacity of lettuce.

N stress significantly impacts plant photosynthetic efficiency. Under N limitation, both photosystem I (PSI) and photosystem II (PSII) undergo photoinhibition, resulting in decreased electron transport rates and subsequent ROS accumulation [39]. In tea plants, N deficiency can decrease the chlorophyll content [40]. This research showed that compared to the WT, the chlorophyll content of transgenic lines was increased, indicating that OsNRT2.3b improved the photosynthetic capacity of lettuce to a certain degree. However, chlorophyll content exhibited only subtle variations under LN treatment relative to the CK, which warrants further investigation.

N serves as a fundamental building block for numerous vital plant compounds [41]. To incorporate N into organic molecules, plants undergo a two-stage biochemical transformation process. Initially, cytoplasmic NR catalyzes the conversion of NO_3_^-^ to NO_2_^-^, which is subsequently reduced to NH_4_^+^ by plastid-localized NiR in both chloroplasts and roots [42,43]. Notably, NO_3_^-^ availability serves as the primary regulator of both NR and NiR enzymatic activities [44]. As transcriptional repressors, LBD37/38/39 directly bind to the promoter of *NiR* to suppress their transcription. LN causes a drastic decline in endogenous Gln content and subsequent downregulation of *LBD37*/*38*/*39*, relieving the inhibitory effect on *NiR* transcription and stimulating NiR accumulation [45]. In this study, LN treatment reduced NO_3_^-^ content and NR activity but increased NiR activity and NH_4_^+^ content. These observations suggest that LN restricted external NO_3_^-^ availability, limiting NR substrate and lowering NR activity as well as NO_3_^-^ content. De-repression at the transcriptional level induced massive NiR synthesis; the sparse remaining NO_2_^-^ was efficiently reduced to NH_4_^+^ by abundant NiR, causing rises in both NiR activity and endogenous ammonium. Furthermore, compared to the WT, *OsNRT2.3b* transgenosis substantially enhanced the NR and NiR activities of lettuce, as well as NO_3_^-^ and NH_4_^+^ contents. This implies that OsNRT2.3b promoted NO_3_^-^ absorption in lettuce, thereby improving NR and NiR activities and facilitating the synthesis of NH_4_^+^.

The assimilation of NH_4_^+^ in plants involves two key enzymatic reactions. First, glutamine synthetase (GS) mediates the ATP-dependent conversion of NH_4_^+^ and glutamate to glutamine in both cytoplasmic and plastid compartments. This is followed by glutamate synthase (GOGAT)-catalyzed reductive amination, where glutamine reacts with α-ketoglutarate to regenerate two glutamate molecules in chloroplasts and root plastids [42,43]. In this study, compared to the WT, the GS and GOGAT activities of transgenic lines were improved. Moreover, soluble proteins and amino acids, the fundamental unit that makes up proteins, were also moderately increased. It can be inferred that the improvement of lettuce NH_4_^+^ enhanced GS and GOGAT activities, thus promoting the synthesis of proteins and amino acids to a certain degree.

Although overexpression of *OsNRT2.3b* enhanced the N utilization efficiency in lettuce, systematic measurements revealed no statistically significant differences in multiple agronomic traits between the transgenic lines and WT. This negative result actually provides counter-evidence: the yield enhancement by *OsNRT2.3b* is not attained through modifications in the fundamental plant architecture or developmental pattern of lettuce, but rather, it is more likely to operate by optimizing physiological processes such as N uptake and translocation. Concurrently, this also suggests that overexpression of this gene exhibits favorable safety profiles, without exerting unintended pleiotropic effects on agronomic traits.

In the future, the functional mechanism of *OsNRT2.3b* in lettuce will be studied further. Simultaneously, we will consider adding multiple N concentration gradients to strengthen the conclusions about N use efficiency.

## Conclusions

In this study, *OsNRT2.3b* was transformed into wild-type (WT) lettuce (Italy). Then, T3 homozygous lines (OE20−5, OE21−7, OE21−11, and OE21−13) and the WT were subjected to normal (CK) and low nitrogen (LN) treatments for 12 d. The results indicate that OsNRT2.3b improves antioxidant enzyme activities, glutathione (GSH) level, chlorophyll content, and N use efficiency in lettuce. In addition, compared to CK, *OsNRT2.3b* can respond to LN stress in transgenic lettuce. In summary, OsNRT2.3b enhances the antioxidant activity, photosynthetic efficiency, and N use efficiency of lettuce. These findings provide theoretical references for optimizing N utilization and stress tolerance in lettuce, with further experimental validation to follow.

## Supporting information

S1 Table Primer sequences in this study. (DOCX)

S2 Table Kits for determining physiological indices. (DOCX)

S1 Fig Gel electrophoregram of verifing the inserted fragment. (PDF)
