# Supplementary material for: Effects of OsNRT2.3b transgenosis on lettuce antioxidant capacity and nitrogen metabolism under low nitrogen
Source: PLoS One. 2026 Jul 1;21(7):e0352238. doi: 10.1371/journal.pone.0352238 (PMC13322504; doi:10.1371/journal.pone.0352238)
Supplement: S1 Table — (DOCX) [file pone.0352238.s001.docx]

**Supplementary Table S1. Primer sequences in this study.**

| Usage | Primer name | Primer sequence (5’-3’) |
| --- | --- | --- |
| Cloning of CDS sequence of *OsNRT2.3b* | LC-OsNRT2.3b-F | CACGCTCGAGCTCAAGCTTGAGCGCCGGCCTCCCACCGG |
|  | LC-OsNRT2.3b-R | TCGCGGCCCCGGTGGATCCCGCATCAAAAAGTTTTGACT |
| Verifcation of the inserted fragment | OsNRT2.3b-F | CGTCATGCTCATCTACTTCCC |
|  | LC-R | TAACGGGTGATATATTCATTAG |
| Quantitative real-time PCR | q-OsNRT2.3b-F | ACAAAGTACAAGACGGAGACC |
|  | q-OsNRT2.3b-R | GAAGTAGATGAGCATGACGGG |

Note: Letters above lines and primer LC-R are carrier sequences.
