# Supplementary material for: Effects of OsNRT2.3b transgenosis on lettuce antioxidant capacity and nitrogen metabolism under low nitrogen
Source: PLoS One. 2026 Jul 1;21(7):e0352238. doi: 10.1371/journal.pone.0352238 (PMC13322504; doi:10.1371/journal.pone.0352238)
Supplement: S2 Table — (DOCX) [file pone.0352238.s002.docx]

**Supplementary Table S2. Kits for determining physiological indices.**

| Kit name | Manufacturer | Catalogue number |
| --- | --- | --- |
| Superoxide Dismutase (SOD)Activity Assay Kit | Solarbio, Beijing, China | BC0175 |
| Peroxidase (POD)Activity Assay Kit |  | BC0095 |
| Catalase (CAT)Activity Assay Kit |  | BC0205 |
| Malondialdehyde (MDA)Content Assay Kit |  | BC0025 |
| Reduced Glutathione (GSH)Content Assay Kit |  | BC1175 |
| Plant Nitrate Nitrogen (NO_3_^-^)Content Assay Kit |  | BC1505 |
| Plant Ammonium Nitrogen Content Assay Kit |  | BC6045 |
| Micro Amino Acid (AA)Content Assay Kit |  | BC1575 |
| Protein Content Assay Kit (Biuret Method) |  | BC3185 |
| Nitrate Reductase (NR)Activity Assay Kit |  | BC0085 |
| NiR Assay Kit |  | BC1545 |
| Glutamine Synthetase (GS)Activity Assay Kit |  | BC0915 |
| Glutamate Synthase (GOGAT)Activity Assay Kit |  | BC0075 |
