# Supplementary figures and images for: Effects of OsNRT2.3b transgenosis on lettuce antioxidant capacity and nitrogen metabolism under low nitrogen
Source: PLoS One. 2026 Jul 1;21(7):e0352238. doi: 10.1371/journal.pone.0352238 (PMC13322504; doi:10.1371/journal.pone.0352238)

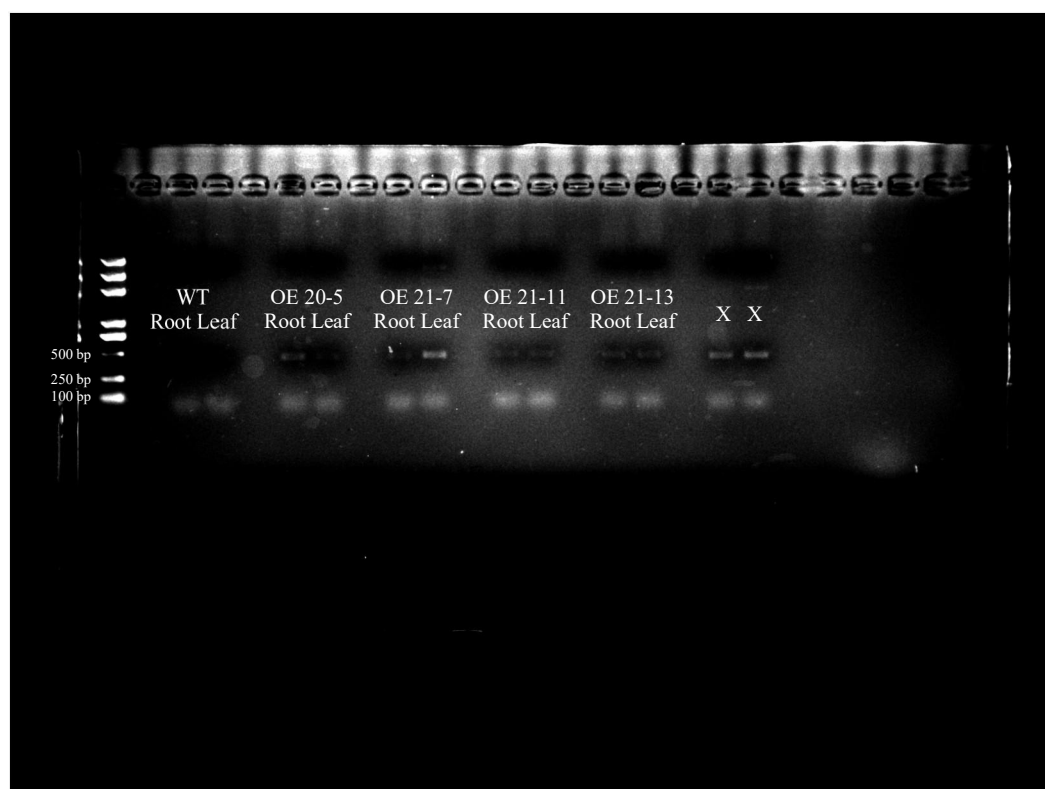

**Fig S1\_raw\_images.** Image Lab 5.2.1 was used to capture the image.

Supplement: S1 Fig — (PDF) [file pone.0352238.s003.pdf]
